# Recommendations to improve insurance coverage for physiotherapy services in Iran: a multi criteria decision-making approach

**DOI:** 10.1186/s12962-021-00333-0

**Published:** 2021-12-11

**Authors:** Saeed Shahabi, Dimitrios Skempes, Masoud Behzadifar, Reza Tabrizi, Behrooz Nazari, Mahboubeh Khaton Ghanbari, Seyed Taghi Heydari, Leila Zarei, Kamran Bagheri Lankarani

**Affiliations:** 1 Health Policy Research Center, Institute of Health, Shiraz University of Medical Sciences, Shiraz, Iran; 2 Independent Researcher, Zofingen, Switzerland; 3 Social Determinants of Health Research Center, Lorestan University of Medical Sciences, Khorramabad, Iran; 4 Noncommunicable Diseases Research Center, Fasa University of Medical Sciences, Fasa, Iran; 5 Rehabilitation Research Center, Department of Physiotherapy, School of Rehabilitation Sciences, Iran University of Medical Sciences, Tehran, Iran; 6 Health Management and Economics Research Center, Iran University of Medical Sciences, Tehran, Iran; 7 Clinical Research Development Unit, Vali Asr Hospital, Fasa University of Medical Sciences, Fasa, Iran

**Keywords:** Insurance coverage, Physiotherapy, Health policy, Rehabilitation, Analytical hierarchy process

## Abstract

**Background:**

High toll of traffic-related injuries, climate change, natural disasters, population aging, as well as chronic diseases have all made considerable demands on receiving physiotherapy services in Iran. Nevertheless, there is an assortment of complications facing utilization of such services, particularly poor insurance coverage. Therefore, the present study investigated and identified gaps in insurance coverage in order to inform future policy reforms and the design of a more comprehensive and universal benefits package for physiotherapy services in Iran.

**Methods:**

This project was carried out in Iran, using a mix-methods (viz. qualitative-quantitative) approach. Within the first phase, a qualitative study was completed to find policy recommendations. Such recommendations were then prioritized through the Analytical Hierarchy Process (AHP), in the second phase, based on effectiveness, acceptability, cost, fairness, feasibility, and time.

**Results:**

Within the first phase, a total number of 30 semi-structured interviews with health policy-makers, health insurers, faculty members, rehabilitation experts, and physiotherapists were completed. Several policy recommendations were also proposed by the study participants. Following the second phase, prioritized recommendations were provided to promote stewardship (e.g., informing policy-makers about physiotherapy services), collection of funds (e.g., placing value-added taxes on luxury goods and services), pooling of funds (e.g., moving allocated resources towards insurance (viz. third-party) mechanism), purchasing (e.g., using strategic purchasing), and benefit package (e.g., considering preventive interventions) as the main components of insurance coverage.

**Conclusion:**

The study findings provided a favorable ground to improve insurance coverage for physiotherapy services in Iran. As well, decision- and policy-makers can place these recommendations on the agenda in the health sector to protect population health status, especially that of groups with disabilities.

**Supplementary Information:**

The online version contains supplementary material available at 10.1186/s12962-021-00333-0.

## Background

Approximately 15% of people worldwide are living with disabilities, predicted to deteriorate in the future years due to high prevalence rates of chronic diseases, population aging, traffic-related injuries, and number of survivors of traumatic events [1]. In this respect, a study in 2019, had further established that the burden of musculoskeletal disorders (MSDs) had significantly multiplied over the past decades, and such problems had been introduced as the second cause of years lived with disability (YLD) [2]. In addition, the growing incidence of neurological disorders such as tetanus, meningitis, encephalitis, stroke, traumatic brain injury (TBI), and spinal cord injury (SCI) in recent years has remained as the main cause of disability-adjusted life years (DALYs) [3]. Low-back pain (LBP), neck pain, osteoarthritis (OA), and rheumatoid arthritis (RA) are also among the leading MSDs, indicating a high burden especially in the Eastern Mediterranean region (EMR) including Iran [4]. Therefore, in accordance with the current global trends, the need for physical rehabilitation services including physiotherapy interventions is on the rise [5].

Given the high rate of traffic-related injuries (e.g., head trauma, fractures, and SCI), climate change, natural disasters, population aging, and chronic diseases, demands for receiving physiotherapy services is also considerable in Iran [6–8]. Therefore, appropriate funding and delivery of these services are of utmost importance. However, physiotherapy services are often regarded as luxury and tertiary ones in Iran's health care system [9]. Most of such services are provided by private centers, and out-of-pocket (OOP) payments are the leading reimbursement mechanisms attributable to insufficient insurance coverage. As a result, a major proportion of households are suffering from catastrophic expenditures (CEs) [10].

Different countries, principally developed ones, have adopted various approaches to cover physiotherapy services within health insurance programs. For instance, physiotherapy services for children up to the age of 18 years are included in the basic health insurance package in the Netherlands; however, there are limitations to utilizing such services for other groups [11]. Furthermore, physiotherapy services are among those covered by statutory health insurance (SHI), consisting of a variety of insurance schemes for the population living in France [12]. Nonetheless, reimbursement is subject to the doctors' prescriptions as well as approval of consulting doctors in the SHI Medical Service Office [12]. In Norway, physiotherapy services are provided at both primary and secondary levels, respectively funded by municipalities and the Norwegian Health Economics Administration (Helseøkonomiforvaltningen: HELFO) [13]. In general, a share of the costs is paid directly by service recipients.

The World Health Organization (WHO) has further introduced rehabilitation services as one of the main dimensions of the universal health coverage (UHC) [14]. Recently, the “WHO Global Disability Action Plan 2014–2021” has been also initiated to meet the increased demands for these interventions [1]. In addition, “Rehabilitation 2030: A Call for Action” has been started to enhance the accessibility of rehabilitation services such as physiotherapy [15]. Integrating rehabilitation services in health care systems and improving insurance coverage for such services are the main goals of this guidance. However, in many countries (especially undeveloped and developing ones), no specific funding has been thus far allotted to the rehabilitation sector [16].

Besides, the UHC aims to provide all populations with the interventions they need at a cost level protecting them from financial hardships [17]. Consequently, financing is one of the key functions in health care systems including physiotherapy services. To secure financial protection, a number of strategies such as pre-payment and pooling are being recommended [18]. Indeed, evidence shows that insurance mechanisms (as a pre-payment approach) facilitate sharing and pooling risks, and ultimately reduce direct payments [19]. Therefore, moving towards an insurance mechanism is the inevitable option to finance health care services such as physiotherapy. Even if preventive, curative, and rehabilitative effects of physiotherapy interventions have been so far confirmed by relevant evidence [20–23], their insurance coverage still low in Iran like many other countries [10]. Nowadays, fragmentation in financing and provision of physiotherapy services, like other Iranian health sub-systems, is leading to unnecessary duplication. In fact, different actors and stakeholders (including the Ministry of Health and Medical Education: MoHME, Social Security Organization: SSO, Iran Health Insurance Organization: IHIO, Armed Forces Social Security Organization: AFSSO, the Iranian Red Crescent Society: IRCS, the State Welfare Organization of Iran, and the Iranian Physiotherapy Association, etc.) are involved in this process [24, 25].

In response to the aforementioned situation, a study was conducted to identify gaps in insurance coverage in order to inform future policy reforms and the design of a more comprehensive and universal benefits package for physiotherapy services in Iran.

## Methods

**Fig. 1:**
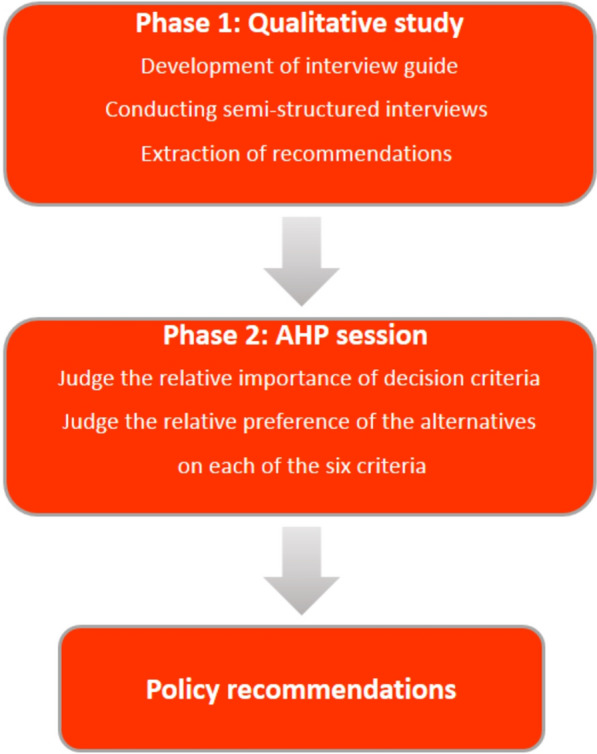
Overview of study methods

The present project was carried out in two phases using a mix-methods approach in Iran encompassing both qualitative and quantitative data collection methods. An overview of the study methodology is outlined in Fig. 1.

### Phase 1

Firstly, an interview study was carried out to obtain the views of key stakeholders on how to improve insurance coverage for physiotherapy services in Iran. The face-to-face semi-structured interviews were accordingly conducted by the first author (a male PhD and health policy-maker experienced in rehabilitation research) in the city of Tehran, the capital of Iran. Besides, Skype and telephone calls were employed to interview those living in other areas. The study participants were also recruited using purposive and snowball sampling methods, and the sampling continued until saturation was achieved. Two interviews with duplicate data were further considered to confirm saturation. It should be noted that the first researcher contacted each participant (via phone calls or e-mails) to set a date and time for the interview session. The study population included health policy-makers, health insurers, faculty members, rehabilitation experts, and physiotherapists (Table 1). Interview guides also consisted of open-ended questions developed based on the conceptual framework components (stewardship, collection of funds, pooling of funds, purchasing, and benefit package) (Additional file 1: Fig. S1) [19]. The interviews also lasted between 20 and 55 min and recorded digitally. Verbatim transcription was further done after each session and the participants were allowed to review their interview transcripts, and if required, they had the opportunity to correct them. Framework analysis was similarly adopted to analyze the collected data. In accordance with this approach, five stages including: (1) familiarizing with collected data; (2) recognizing thematic framework; (3) indexing; (4) charting; and (5) mapping and interpreting were considered [26]. In addition, peer debriefing, triangulation, as well as prolonged engagement of the first author, were taken into account to enhance rigor and trustworthiness [27]. To ensure the participants' anonymity, a series of ID numbers were used throughout the transcriptions.

**Table 1:** Demographic characteristics of participants

ID	Gender	Professional experience (years)	Specialty	Workplace
Health policy-maker 1	Male	14	Health policy	MoHME
Health policy-maker 2	Male	11	Health policy	MoHME
Health policy-maker 3	Female	7	Health management	IRCS
Health policy-maker 4	Male	9	Health policy	Medical university
Health policy-maker 5	Male	21	Health financing	PBO
Health policy-maker 6	Female	8	Health financing	IHIO
Health policy-maker 7	Female	7	Health insurance	IHIO
Health policy-maker 8	Male	7	Health insurance	SSO
Health policy-maker 9	Female	5	Health management	SSO
Health policy-maker 10	Male	12	Health policy	Medical University
Rehabilitation manager 1	Female	8	Physiotherapy	Welfare Organization
Rehabilitation manager 2	Male	10	Medical doctor	Welfare Organization
Rehabilitation manager 3	Male	28	Occupational therapy	Private
Rehabilitation manager 4	Male	6	Medical doctor	IRCS
Rehabilitation manager 5	Female	11	Physiotherapy	Private
University professor 1	Female	5	Physiotherapy	Medical University
University professor 2	Male	5	Physiotherapy	Medical University
University professor 3	Male	18	Physiotherapy	Medical University
University professor 4	Female	13	Physiotherapy	Medical University
University professor 5	Male	6	Physiotherapy	Medical University
University professor 6	Male	6	Physiotherapy	Medical University
University professor 7	Female	5	Physiotherapy	Medical University
Physiotherapist 1	Male	5	Physiotherapy	Public
Physiotherapist 2	Male	9	Physiotherapy	Public
Physiotherapist 3	Female	17	Physiotherapy	Private
Physiotherapist 4	Female	6	Physiotherapy	Public
Physiotherapist 5	Male	8	Physiotherapy	Private
Physiotherapist 6	Female	5	Physiotherapy	Private
Physiotherapist 7	Male	19	Physiotherapy	Public
Physiotherapist 8	Male	10	Physiotherapy	Private

*MoHME* Ministry of Health and Medical Education; *IRCS* Iranian Red Crescent Society; *PBO* Budget and Planning Organization; *IHIO* Iran Health Insurance Organization; *SSO* Social Security Organization

### Phase 2

**Fig. 2:**
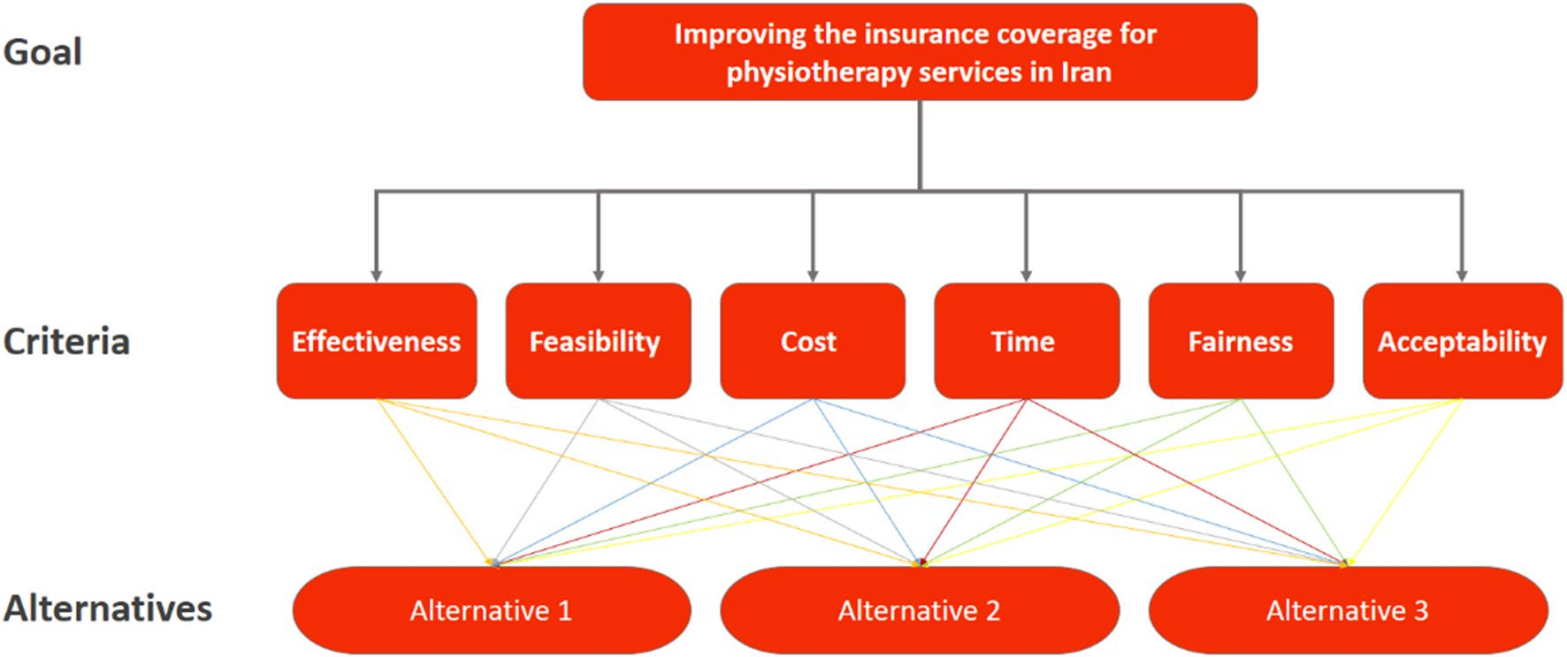
AHP hierarchy which represents the main goal, interested criteria, and potential alternatives (recommendations)

In the second phase, the analytical hierarchy process (AHP), developed by Dr. Saaty in 1977, was employed to prioritize the policy recommendations obtained [28]. AHP is a multi criteria decision-making (MCDM) approach that uses pairwise comparisons to compare available alternatives with relevant criteria and to determine the best ones (Fig. 2). Based on the WHO priority-setting guideline, six criteria were selected as most significant for the study: effectiveness, acceptability, cost, fairness, feasibility, and time [29]. The relative importance of these six criteria was also obtained from 11 experts (by S.Sh), either via e-mails or at their workplace. The experts who participated in the weighting of the criteria had diverse academic and professional backgrounds, specifically experts included three university professors, three licensed physiotherapists, two health policy-makers, and three health financing officers. Regarding the sample size, there are no pre-defined guidelines as to how many experts should participate in the AHP. The sample size will largely depend on the study aim, and in general, this approach does not need a large sample [30]. In fact, one expert's viewpoint may suffice, in accordance with the aims of the study, unless several experts from different backgrounds are necessary, and so, various experts are needed if they are accessible [31]. With reference to the pairwise comparison matrix, experts were asked to express their viewpoints using Saaty’s nine-point rating scale (Table 2). Then, the final value for each pairwise comparison was calculated based on the geometric-logarithmic mean. These weighted values revealed the relative importance of each criterion, and these values were utilized to determine relative preferences for the recommendations. Furthermore, the inconsistency rate (IR) of the experts' viewpoints was calculated for each pairwise comparison. In accordance with the evidence, an IR less than or equal to 0.1 (IR  =   < 0.10) could be accepted [32]. The AHP analysis was also conducted using the Expert Choice (EC) 11 software (Arlington, Virginia, USA).

**Table 2:** Saaty’s pairwise comparison rating

Degree of importance	Level of preference	Description
1	Equal	Two criteria contribute equally to the goal
2	Weak	
3	Moderate	Judgment slightly to moderately in favor of the one criterion against another
4	Moderate plus	
5	Strong	Judgment strongly in favor of the one criterion against another
6	Strong plus	
7	Very strong	Judgment strongly in favor of the one criterion against another and its dominance is confirmed in practice
8	Very, very strong	
9	Extreme	Judgment highest degree possible in favor of the one criterion against another

The study received ethical approval by the Ethics Committee of the National Institute for Medical Research Development (NIMAD), Tehran, Iran (IR.NIMAD.REC.1398.337).

## Results

All through the qualitative study, the participants expressed a number of policy recommendations for each component of the conceptual framework, as shown in Table 3. Stewardship including inter-sectoral leadership and intra-sectoral governance was thus highlighted by most of the participants. Accordingly, they proposed several options to improve this domain such as moving towards united stewardship, informing policy-makers about physiotherapy services and their effects, involving rehabilitation experts in decision- and policy-making processes, etc. (Table 3a). Enhancing funds or revenue collection was another main component of the financing process with various policy solutions recommended by the study participants especially policy-makers and faculty members such as levying value-added taxes on luxury goods and services, considering higher insurance contributions (i.e., premiums) for childless families, earmarking allocated resources, etc. (Table 3b). Other policy recommendations to boost insurance coverage for physiotherapy services such as pooling of funds, purchasing, and benefit package are respectively illustrated in Table 3c–e.

**Table 3:** Recommendations to improve the insurance coverage for physiotherapy services

IDs	Recommendations	Quotes
(a) Stewardship		
1	Moving towards united stewardship	“A variety of organizations and institutions are currently involved in providing and financing rehabilitation services, including physiotherapy ones in Iran. Well, that is why there are many fragmentations. Moving towards a united stewardship can curb redundancies and waste”. [HPM2]
2	Informing policy-makers about physiotherapy services and their effects	“You know that many decision- and policy-makers in our health care system are not fully aware of physiotherapy services. I think efforts should be made to inform these people”. [UP5]
3	Involving rehabilitation experts in decision- and policy-making processes	“More involvement of physiotherapists and relevant experts in policy-making processes can lead to the promotion of these services in the health care system”. [RM3]
4	Improving HTA process	“Today, in countries such as the United Kingdom, the use of health technology assessment is a prerequisite for considering health interventions in benefit health packages. Therefore, it is necessary to use this approach to cover physiotherapy services by health insurance”. [HPM6]
5	Facilitating interdisciplinary collaborations	“One of our serious challenges in the service delivery process is poor collaboration with other health professionals and even rehabilitation professionals, which has left them with insufficient information about our services”. [PT1]
6	Conducting regular supervisions and accreditations	“The quality of provided services cannot be guaranteed unless there is comprehensive and proper supervision of physiotherapy centers”. [RM1]
7	Strengthening referral systems	“All patients should be now referred to us by a physician. Well … due to our poor referral systems, we cannot expect patients come on time”. [PT7]
8	Conducting comprehensive needs assessments	“The importance of rehabilitation services such as physiotherapy becomes evident to policy-makers when the population requesting these services is determined. Therefore, we need to move forward by conducting needs assessments at the national and regional levels”. [RM1]
9	Establishing accurate information systems	“Existence of accurate information systems such as electronic files can facilitate awareness of the services provided to patients and their effects. As a result, the insurer can cover the services”. [UP4]
10	Prioritizing and rationing	“As there is no clear prioritization process in Iran, many expensive services are included in the basic packages by influential groups, and services such as physiotherapy, which have less influence and power, are being excluded”. [RM3]
11	Promoting interactions between scientific associations and insurers	“The Iranian Physiotherapy Association can persuade other financing agencies, such as insurance companies, to cover physiotherapy services”. [PT3]
(b) Collection of funds		
1	Levying value-added taxes on luxury goods and services	“One of the appropriate ways to finance rehabilitation services such as physiotherapy in Iran is to impose a taxes on luxury goods”. [HPM7]
2	Placing taxes on chemical and toxic industries	“You see, chemical and oil industries are one of the causes of chronic diseases and disabilities in Iran due to pollution, so it is possible to provide necessary resources for insurance coverage by imposing special taxes on these industries”. [RM5]
3	Imposing taxes on harmful substances such as tobacco, sugar, etc.	“Many disabilities and illnesses, such as stroke, in which people need physiotherapy services, are caused by harmful substances such as smoking. Therefore, taxing these materials can facilitate financing”. [UP2]
4	Considering higher insurance contributions for childless families	“Some developed countries charge higher premiums from childless families because they need more rehabilitation in times of disability and old age they need more rehabilitation”. [UP4]
5	Obligating complementary health insurance for employed groups	“In my opinion, we should make supplementary insurance mandatory for some working groups. This process will facilitate the coverage of insurance services such as physiotherapy ones”. [HPM6]
6	Promoting rehabilitation funding proportion from public budget	“The share of rehabilitation services in the government budget allocated to the health system is very small. Therefore, like many countries, the government’s share of financing these services must be increased”. [RM4]
7	Earmarking allocated resources	“Although some government resources are allocated to finance rehabilitation services such as physiotherapy, since they are not marked, they are not well allocated to provide these services”. [HPM6]
8	Considering a separate premium for rehabilitation services (including physiotherapy)	“Rehabilitation services are expensive and that is why many insurance companies are reluctant to cover them. One solution would be to set a separate premium for these services”. [UP3]
(c) Pooling of funds		
1	Moving allocated resources towards insurance (i.e., third party) mechanisms	“Incorporating funds into the insurance process can reduce financial pressures on high-risk groups by sharing risks”. [HPM1]
2	Integrating insurance funds	“Existence of multiple insurance funds in the Iranian health system is one of the major financing challenges. Merging and integration of these funds can be thus a potential solution”. [UP6]
3	Cross-subsiding across different regions and groups at national level	“Given the differences between regions as well as different demographic groups, cross subsidies at the national level can be an appropriate policy solution to reduce injustice”. [HPM7]
4	Using individual medical saving accounts	“One of the interesting solutions in health financing system is individual medical saving accounts in Singapore. This approach can be also used to finance physiotherapy services, as it increases the responsibility of individuals and also divides the risks over time”. [HPM7]
5	Consolidating small insurance funds	“There are several insurance funds in Iran that are very small. For this reason, they are not productive enough. So, consolidating these funds can be a good approach”. [HPM2]
(d) Purchasing		
1	Separating providers and purchasers	“Interestingly, the Ministry of Health and Medical Education is both the main financer and provider of health services in Iran. However, the separation between the financier and the provider is one of the principles of favorable financing”. [HPM1]
2	Exploiting strategic purchasing	“Coverage of physiotherapy services by health insurers requires the use of strategic purchasing principles”. [HPM6]
3	Considering quality indicators in purchasing process	“I think it is better to buy services based on quality indicators and clinical effects in order to reduce the purchases of poor quality services”. [UP2]
4	Utilizing performance- or outcome-based payment systems	“One of the recent approaches in many developed countries to improve motivation and quality of services is to link payment mechanisms to performance and health outcomes”. [PT5]
5	Limiting induced demands by payment and punishing mechanisms	“I myself have seen several times that service providers deliver services to patients that are not really needed. Well… there must be effective punitive mechanisms”. [UP1]
6	Using rehabilitation experts in purchasing process	“The presence of physiotherapists in the purchasing process, given their familiarity with the services, can prevent many abuses and misbehaviors”. [PT7]
7	Correcting tariffs based on economic situation	“Nowadays, service tariffs are not really compatible with inflation and rising costs”. [RM3]
8	Reducing co-payment rates	“Although some physiotherapy services are covered by health insurers, co-payment by recipients is still high”. [UP2]
9	Practicing capitation mechanisms to control expenditures	“Per capita payment is one of the effective approaches to control costs, especially in primary health care. This mechanism can be also used for physiotherapy services in primary health care”. [HPM7]
(e) Benefit package		
1	Considering cost-effectiveness interventions	“Many physiotherapy services are cost-effective. At least the same services can be covered by insurance”. [UP2]
2	Using preventive interventions	“Interestingly, many physiotherapy services have preventative effects that can thwart majority of severe disabilities”. [UP3]
3	Practicing needed interventions in golden time treatments after diseases	“If physiotherapy interventions are provided early for some patients, such as stroke cases, especially in the first three to six months after their occurrence, they can prevent many long-term complications such as reduced range of motions of the joints”. [PT1]
4	Reflecting on inpatient interventions	“Physiotherapy services required by hospitalized patients can be covered by health insurance to avoid heavy costs. For example, cardiac rehabilitation services can speed up a patient’s recovery and discharge”. [UP5]
5	Covering physiotherapy services required for children up to the age of 6 years	“It is true that health insurance institutions face limited resources, but like many developed countries, physiotherapy services needed by children up to the age of 5 or 6 years can be at least covered in Iran”. [HPM5]
6	Covering of physiotherapy services up to the age of 18 years	“Since many musculoskeletal disorders can be corrected by the time a person reaches puberty, covering physiotherapy services until the age of 18 years can improve public health and avoid heavy costs in the future”. [PT7]

Regarding the AHP session, the findings of the pairwise comparisons of the six criteria are presented in Table 4. In this respect, the relative importance of the criteria included feasibility with a ratio of 0.258, which had the highest importance as well as acceptability, fairness, cost, effectiveness respectively with ratio of 0.178, 0.171, 0.138, and 0.131. Moreover, time with a ratio of 0.124 was given the lowest importance. In the pairwise comparison of the six criteria compared with the goals, the IR was 0.09.

**Table 4:** Matrix of pairwise comparisons

Main criterion	Feasibility	Acceptability	Fairness	Cost	Effectiveness	Time	Relative importance
Feasibility	1	1	2	2	3	2	0.258
Acceptability	1	1	1/2	1	2	2	0.178
Fairness	1/2	2	1	2	1	1/2	0.171
Cost	1/2	1	1/2	1	1	2	0.138
Effectiveness	1/3	1/2	1	1	1	2	0.131
Time	1/2	1/2	2	1/2	1/2	1	0.124

**Fig. 3:**
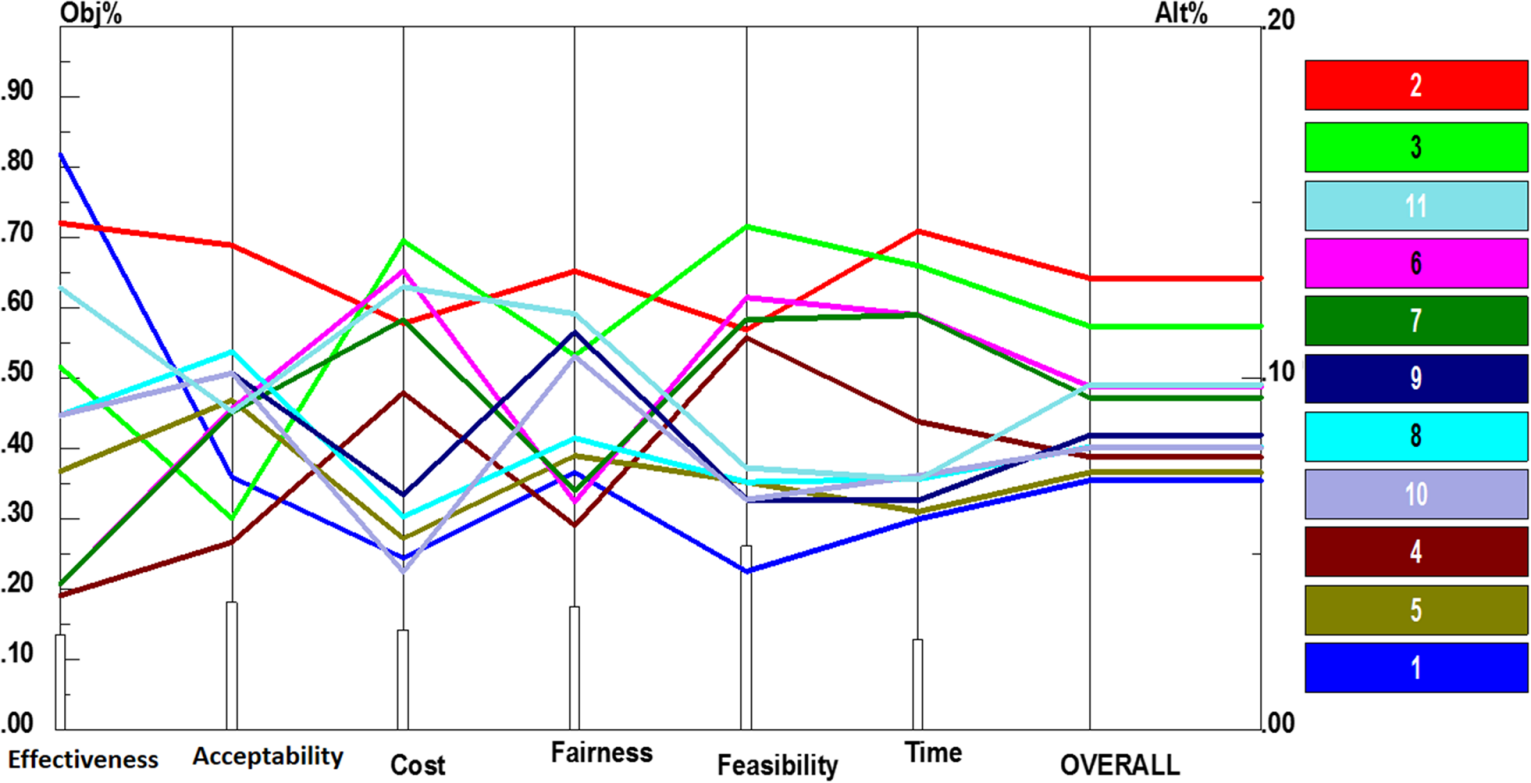
Performance sensitivity analysis of policy recommendations for stewardship based on the six criteria and overall priority

Afterwards, policy recommendations of each component were paired and compared in accordance with the six criteria. Additional file 1: Fig. S2 presents the prioritization of the recommendations for stewardship based on each criterion. According to Fig. 3, informing policy-makers about physiotherapy services and their effects (0.128) obtained the highest priority. It was then followed by involving rehabilitation experts in decision- and policy-making process (0.115), promoting interactions between scientific associations and insurers (0.098), conducting regular supervisions and accreditations (0.098), strengthening referral systems (0.095), as well as establishing accurate information systems (0.084). Moreover, conducting comprehensive needs assessments (0.080), prioritizing and rationing (0.080), improving health technology assessment (HTA) process (0.078), facilitating interdisciplinary collaborations (0.073), and finally moving towards united stewardship (0.071), received the lowest priority. Performance sensitivity analysis of these recommendations also demonstrated in Fig. 3.

**Fig. 4:**
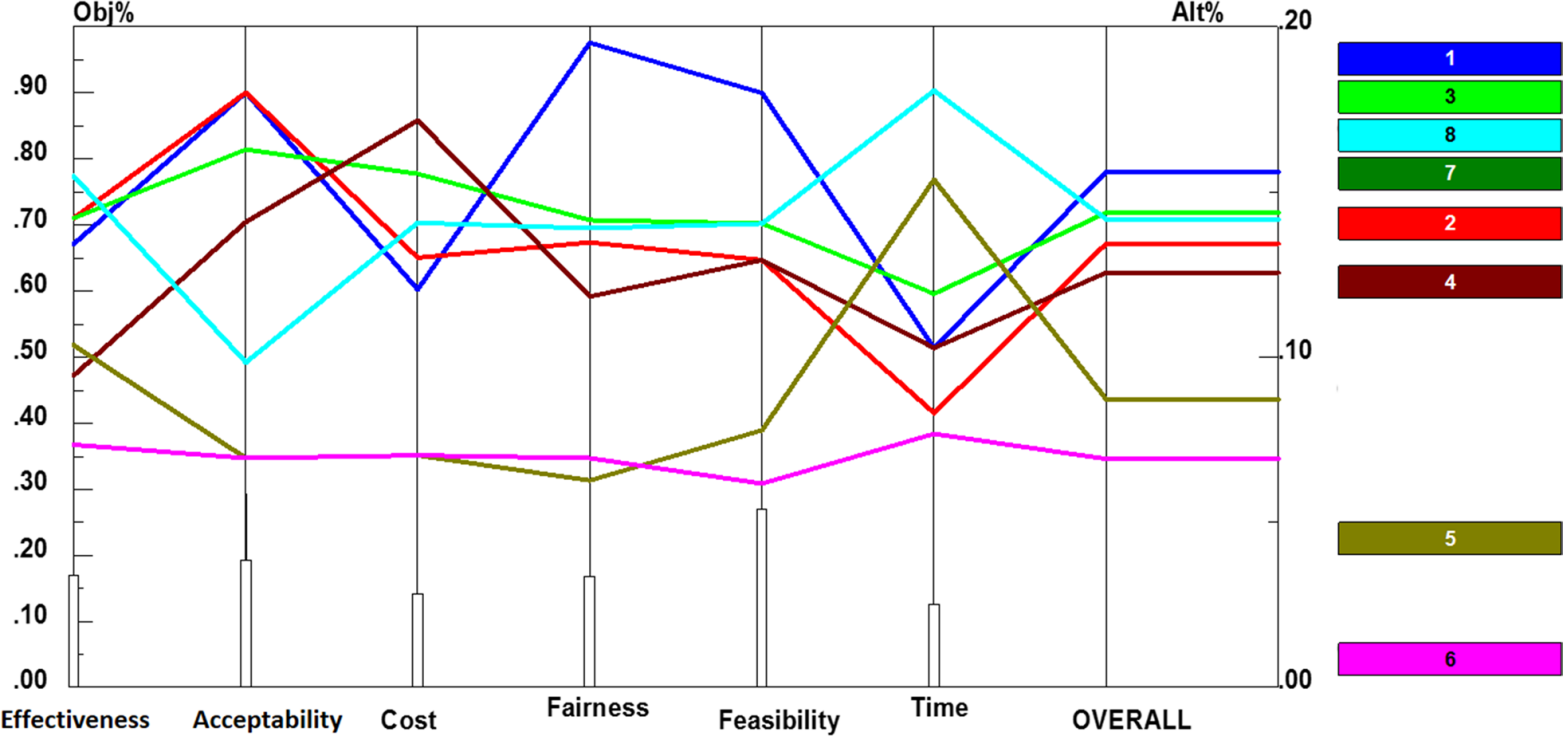
Performance sensitivity analysis of policy recommendations for collection of funds based on the six criteria and overall priority

Prioritization of policy recommendations for collection of funds based on the six criteria are shown in Additional file 1: Fig. S3. In addition, Fig. 4 depicts performance sensitivity analysis and overall prioritization of the recommendations in accordance with the criteria for this component. Accordingly, levying value-added taxes on luxury goods and services (0.158) as well as harmful substances such as tobacco, sugar, etc., (0.144), using a separate premium for rehabilitation services (including physiotherapy) (0.140), earmarking allocated resources (0.140), placing taxes on chemical and toxic industries (0.135), considering higher insurance contributions for childless families (0.127), obligating complementary health insurance for employed groups (0.086), and promoting rehabilitation funding proportion from public budget (0.069), were ranked from the highest to the lowest priority.

**Fig. 5:**
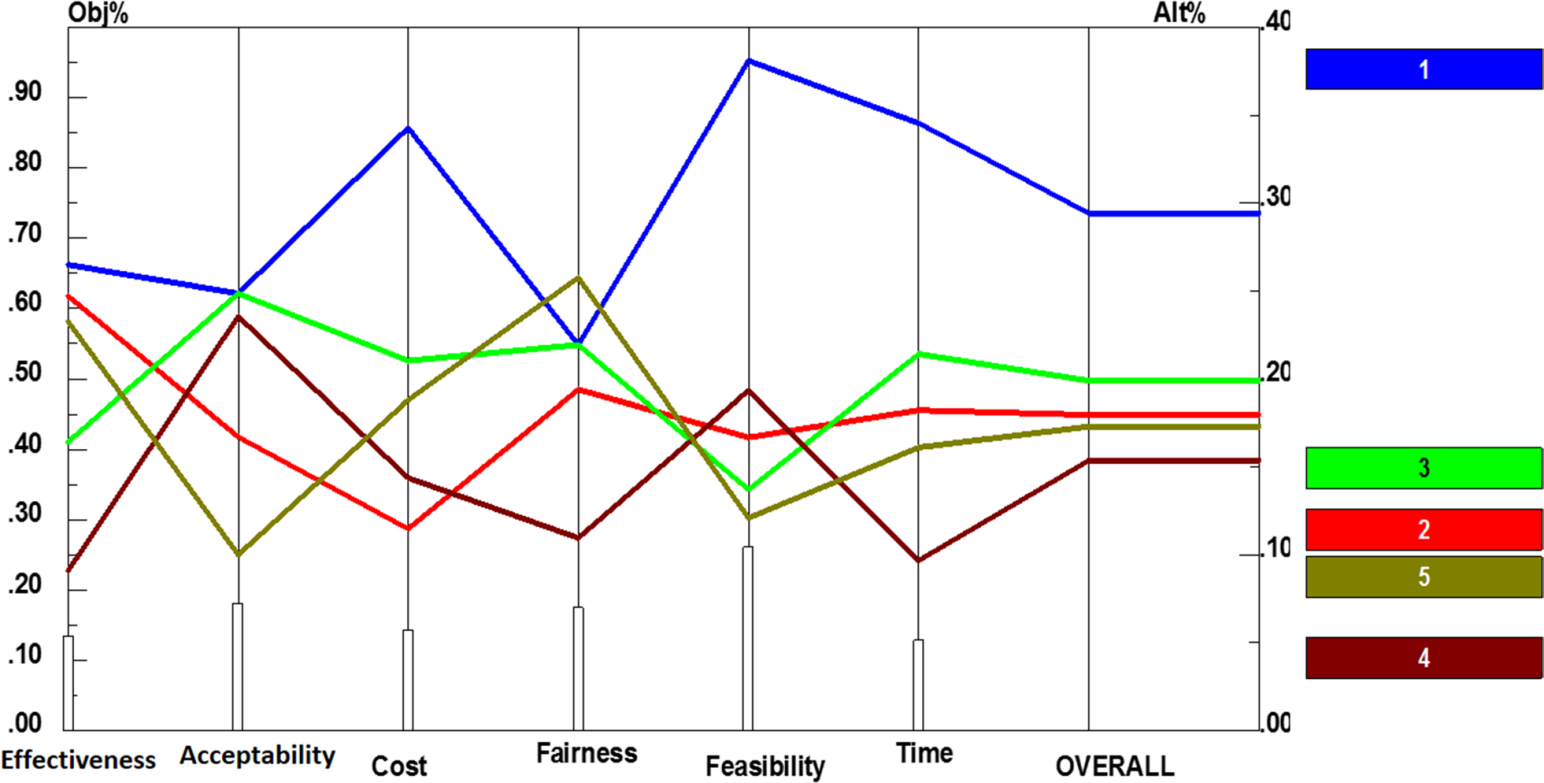
Performance sensitivity analysis of policy recommendations for pooling of funds based on the six criteria and overall priority

Prioritization of policy recommendations for pooling of funds based on the six criteria are also demonstrated in Additional file 1: Fig. S4. As presented in Fig. 5, moving allocated resources towards insurance (i.e., third-party) mechanisms (0.294), had the highest priority, followed by cross-subsiding across different groups at the national level (0.199), integrating insurance funds (0.180), consolidating small insurance funds (0.173), and finally, exploiting individual medical saving accounts (0.154), which had the lowest priority. In addition, performance sensitivity analysis based on each criterion is illustrated in Fig. 5.

**Fig. 6:**
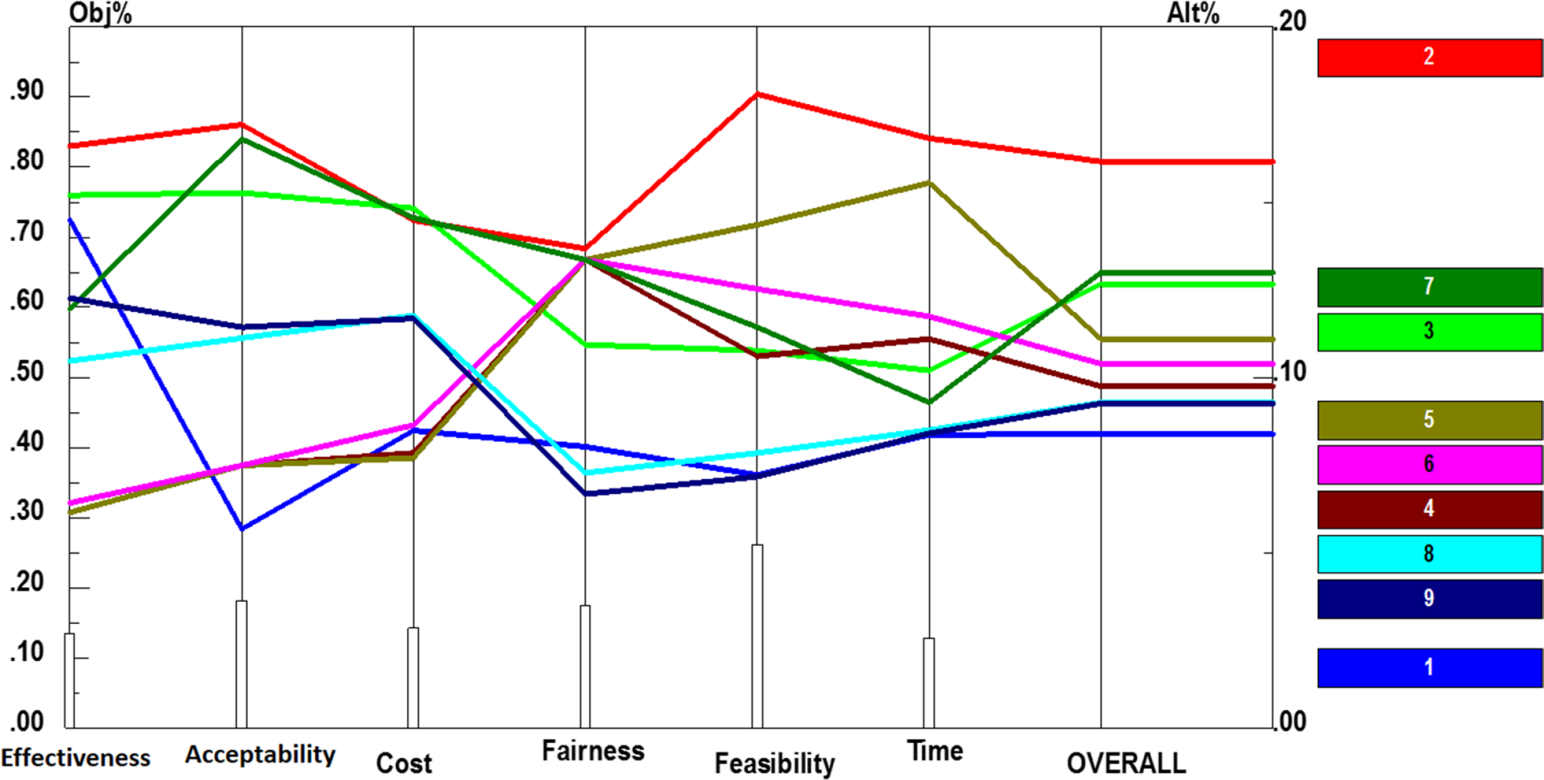
Performance sensitivity analysis of policy recommendations for purchasing based on the six criteria and overall priority

Purchasing was among other components of financing, receiving a number of policy recommendations to improve it. Additional file 1: Fig. S5 shows the prioritization of the obtained alternatives based on the six criteria mentioned. Furthermore, performance sensitivity analysis and overall prioritization are demonstrated in Fig. 6. As shown, using strategic purchasing (0.162), correcting tariffs based on economic situation (0.130), and considering quality indicators in purchasing process (0.127) were the top three priorities. In addition, limiting induced demands by payments and punishing mechanisms (0.111), using rehabilitation experts in purchasing process (0.104), considering performance- or outcome-based payment systems (0.097), reducing co-payment rates (0.093), and exploiting capitation payment mechanisms to control expenditures (0.093), were ranked from four to eight. Finally, separation between provider and purchaser (0.084) had the lowest priority; however, it was one of the top priorities with reference to effectiveness.

**Fig. 7:**
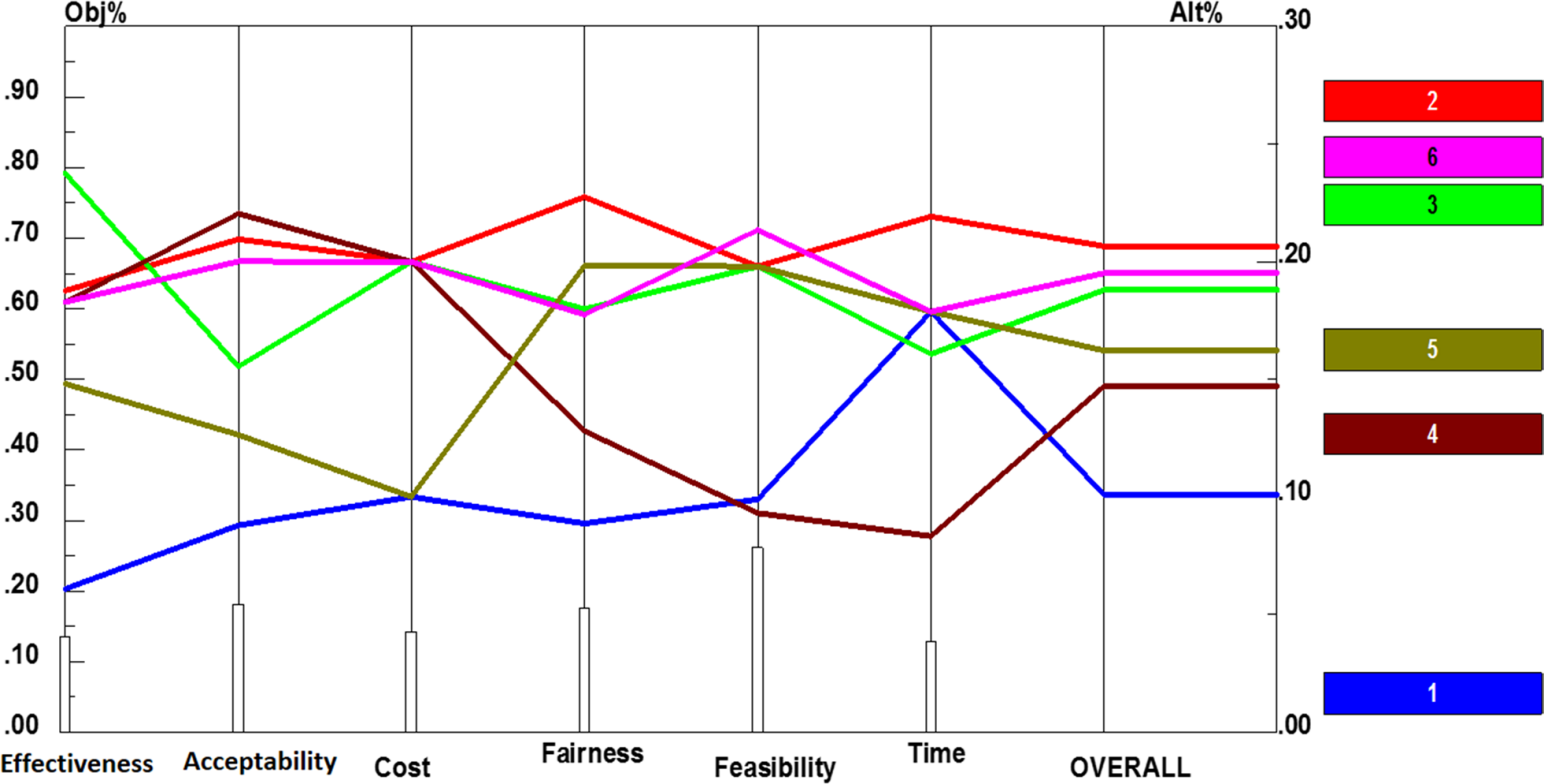
Performance sensitivity analysis of policy recommendations for benefits package based on the six criteria and overall priority

The last component was benefit package, whose policy recommendations were prioritized based on each criterion (Additional file 1: Fig. S6). Like other components, performance sensitivity analysis and overall prioritization of benefit package are described in Fig. 7. The analyses revealed that considering preventive interventions (0.207) had obtained the first rank, which was notable. Furthermore, coverage for physiotherapy services up to the age of 18 years (0.195) and considering interventions required in golden time treatment after diseases (such as stroke) (0.188) were the second and third priorities. Finally, coverage for physiotherapy services required for children up to the age of six years (0.162) along with considering inpatient (0.147) and cost-effectiveness interventions (0.101) were other alternatives to improve the benefit package offered by insurance institutes.

## Discussion

### Stewardship

As a result, raising policy-makers’ awareness may be a significant policy recommendation to strengthen stewardship of financing. Indeed, most of health policy-makers are unaware of these services in Iran [9, 33]. Furthermore, participation of patients and rehabilitation experts in policy processes is another option, which has been noted by recent evidence [34]. Unfortunately, disabled individuals and other relevant groups do not have enough power or influence to get involved in decision- and policy-making processes in Iran [24]. Collaboration between scientific associations and health insurers can be accordingly an alternative to improve insurance coverage for physiotherapy service. Scientific associations can thus provide a list of interventions with lower costs and prevent many future complications. Furthermore, improving the HTA can play an important role in this domain [35]. Even so, weaknesses in HTA process are among common problems facing the Iranian health care system [36]. Therefore, further economic evaluation (i.e., cost-effectiveness, cost-utility, and cost–benefit) should be conducted to demonstrate the effects and the costs of physiotherapy services. Another proposed policy is improving supervision and accreditation systems. Like many other countries, there are unfortunately no transparent and regular supervision systems in the health-related rehabilitation sector including physiotherapy in Iran [24]. In addition, lack of comprehensive accreditation is one other feature of the physiotherapy sector in this country [37]. Despite this fact, providers' accreditation is assumed as a standard tool to assure the quality of services that are very important for insurers [38]. Therefore, developing effective supervisions and accreditations is necessary for the physiotherapy sector. Evidence-based prioritizing and rationing in the health system is thus a proposed recommendation, which can promote the physiotherapy status in benefit packages. However, current health care rationing does not follow any convincing rationales [39, 40]. Fragmentation in stewardship of financing is always shown as one of the significant barriers to universal insurance coverage for health services including physiotherapy in Iran. Consequently, moving towards united stewardship may be a potential policy recommendation [17]. Although the study findings prioritized united stewardship as one of the best alternatives based on effectiveness criterion, it surprisingly had no high priority based on other criteria. Indeed, in accordance with the current situation of Iran, this policy fails to be a feasible and acceptable recommendation.

### Collection of funds

With regard to collection of funds, several recommendations were presented in this study. In this line, the WHO always laid focus on innovative ways such as levying taxes on chemical and toxic industries, placing value-added taxes on luxury goods and services, and imposing taxes on harmful substances such as tobacco, sugar [18, 19] which was accordance with the study findings. These policy options can thus provide new funding resources of insurance coverage for physiotherapy services. Earmarked taxes, also known as hypothecated ones, can be accordingly developed for particular plans [18]. Most of the policy-makers also believed that earmarked taxes could be a considerable fund source for rehabilitation services such as physiotherapy. The ministries of health are often favoring these taxes since they secure financing, especially for health promotion and prevention. Furthermore, the study participants highlighted that approximately all the funds to physiotherapy and other rehabilitation services, should be earmarked. Based on the Fourth Economic, Social, and Cultural Development Plan Act (Article 92) in Iran, 10% of third-party insurance must be allotted to cover medical expenditures of traffic-related injuries [41]. Therefore, as many traffic-related injuries require physiotherapy services, a part of this fund can be also earmarked for physiotherapy services. Considering higher insurance contributions by childless families was another notable policy recommendation for collection of funds. Policy-makers also believed that individuals without any children needed more rehabilitation services such as physiotherapy. Therefore, this premium could provide expedient financial resources.

### Pooling of funds

Pre-payment is the only way to reduce direct payments and financial hardships. In this approach, premiums are collected through insurance mechanisms and then risks are shared and pooled [42]. In agreement with evidence, the study findings concluded that moving the allocated resources for rehabilitation services towards insurance (viz. third party) mechanisms was essential. However, the current funding for rehabilitation services was highly dispersed and each organization was receiving a separate budget [24, 43]. Additionally, using effective third-party mechanisms at the national level facilitates cross-subsidization. This policy is possible wherein multiple funds and different insured groups (namely, poor and rich or young and old) are available [18]. Nevertheless, fragmented funds are one of the key challenges of health care financing in Iran, which work against equity goals [42]. Therefore, integrating insurance funds or consolidating small insurance ones can be among policy options to improve this situation. The participants also discussed that the current fragmentation had reduced efficiency and capacity for cross-subsidization, as mentioned in previous evidence [44, 45].

### Purchasing

Concerning the purchases, several policy recommendations were proposed. Based on prioritization, using strategic purchasing had the highest rank. These results were consistent with the relevant evidence [46, 47]. However, a major proportion of health care services such as physiotherapy are provided by financers. Indeed, there is no actual purchaser-provider split. Therefore, considering strategic purchasing principals such as focus on quality indicators in purchasing process and using performance- or outcome-based payment system can be one of the best alternatives to improve insurance coverage for physiotherapy services. In line with these findings, a recent study in Sweden, England, and the Netherlands had reflected on the importance of strategic purchasing in managing chronic care processes [48]. Furthermore, the study participants believed that current tariffs were not real and needed to be corrected in accordance with economic changes. Recent studies had correspondingly confirmed this problem in financing within Iran's health care system [49, 50]. Therefore, setting real tariffs could increase utilization and prevent informal payments.

### Benefit package

In Iran, basic and complementary health benefit packages are being developed by the High Council of Health Insurance (HCHI) and all health insurance schemes are obligated to follow it [49]. Unfortunately, health-related rehabilitation services including physiotherapy have not been so far well considered. As a result, the number of interventions and the depth of insurance coverage for physiotherapy services are very poor. Despite this, the present study recommended significant policy options to improve this situation. Preventive physiotherapy interventions can be also an attractive alternative for decision- and policy-makers. In fact, many physiotherapy services have preventive effects, which can put a stop to expensive interventions such as surgeries in future [20, 22, 51]. Additionally, some physiotherapy interventions if prescribed until the age of 18 years, can be very effective [52, 53]. In this regard, the Netherlands has included physiotherapy services needed by children up to the age of 18 years in its basic health benefit package [11]. Therefore, considering these interventions and cost-effectiveness strategies can be added to benefit packages [54]. During the interviews, providers and faculty-members also underlined the importance of physiotherapy services after stroke and traumatic events. As shown by evidence, if physiotherapy interventions are provided at golden time (6 months after stroke or 12 months after traumatic injuries), many side effects are thwarted [21, 55]. Therefore, these services may be other potential interventions to be considered by health insurers.

## Study strengths and limitations

The selected face-to-face interview sites in this study were mainly in metropolitan areas, which may limit the generalizability of the findings. However, there were attempts to interview other experts using Skype and telephone calls to deal with this problem. As well, some participants especially health policy-makers had no willingness to participate in this study. Nevertheless, a broad and diverse sample of the participants was selected to minimize this limitation. Lack of participation of patients among included individuals was another limitation of this study. The strength was the participatory and multi-stakeholder approach used which helped elicit the diverse perspectives of clinical professionals and health policy makers was the main strength of the present study.

## Conclusions

In this study, the research team tried to obtain the perspectives of the key stakeholders on how insurance coverage for PT services in Iran can be improved. The findings of this study provide a preliminary evidence base to guide future decisions and reforms aiming to improve insurance coverage for physiotherapy services. Furthermore, decision- and policy-makers may consider including the study's recommendations on current and future health policy in an effort to accelerate progress towards Sustainable Development Goal 3 and UHC, especially for the most vulnerable segments of the population that are frequent users of physiotherapy and rehabilitation services such as people with disabilities.

## Supplementary Information

**Additional file 1: Figure S1.** Conceptual framework. **Figure S2.** Prioritization of recommendations for stewardship based on six criteria. **Figure S3.** Prioritization of recommendations for collection of funds based on six criteria. **Figure S4.** Prioritization of recommendations for pooling of funds based on six criteria. **Figure S5.** Prioritization of recommendations for purchasing based on six criteria. **Figure S6.** Prioritization of recommendations for benefit package on six criteria.

## Data Availability

The data collected and analyzed during the study are available from the corresponding author on reasonable request.

## Abbreviations

MSK: Musculoskeletal
YLDs: Years lived with disability
DALYs: Disability-adjusted life years
LBP: Low-back pain
OA: Osteoarthritis
AR: Arthritis rheumatoid
EMR: Eastern Mediterranean Region
OOP: Out-of-pocket
CEs: Catastrophic expenditures
WHO: World Health Organization
UHC: Universal health coverage
SSO: Social Security Organization
IHIO: Iran Health Insurance Organization
AFSSO: Armed Forces Social Security Organization
AHP: Analytical hierarchy process
MCDM: Multi-criteria decision making
HCHI: High Council of Health Insurance
