# Supplementary material for: Recommendations to improve insurance coverage for physiotherapy services in Iran: a multi criteria decision-making approach
Source: Cost Eff Resour Alloc. 2021 Dec 11;19:80. doi: 10.1186/s12962-021-00333-0 (PMC8666042; doi:10.1186/s12962-021-00333-0)

**Supplemental Fig 1** Conceptual framework


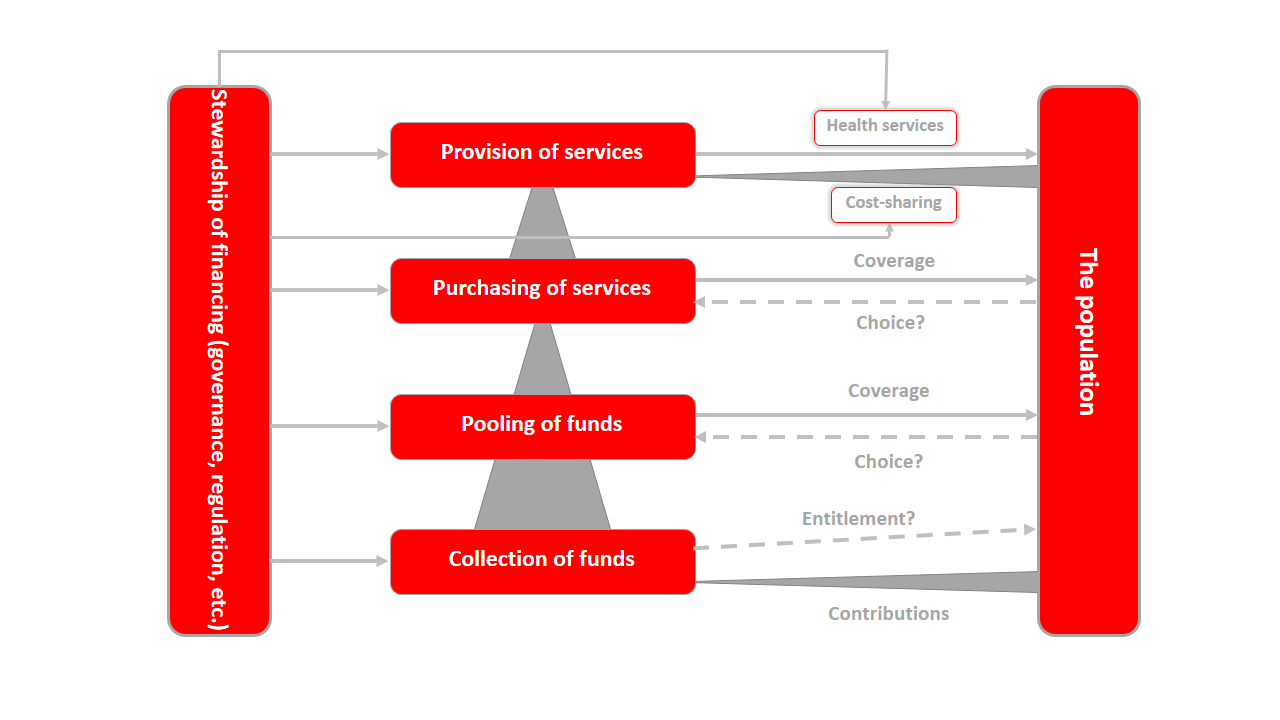


**Supplemental Fig 2** Prioritization of recommendations for stewardship based on six criteria: (a) effectiveness; (b) acceptability; (c) cost; (d) fairness; (e) feasibility; and (f) time.


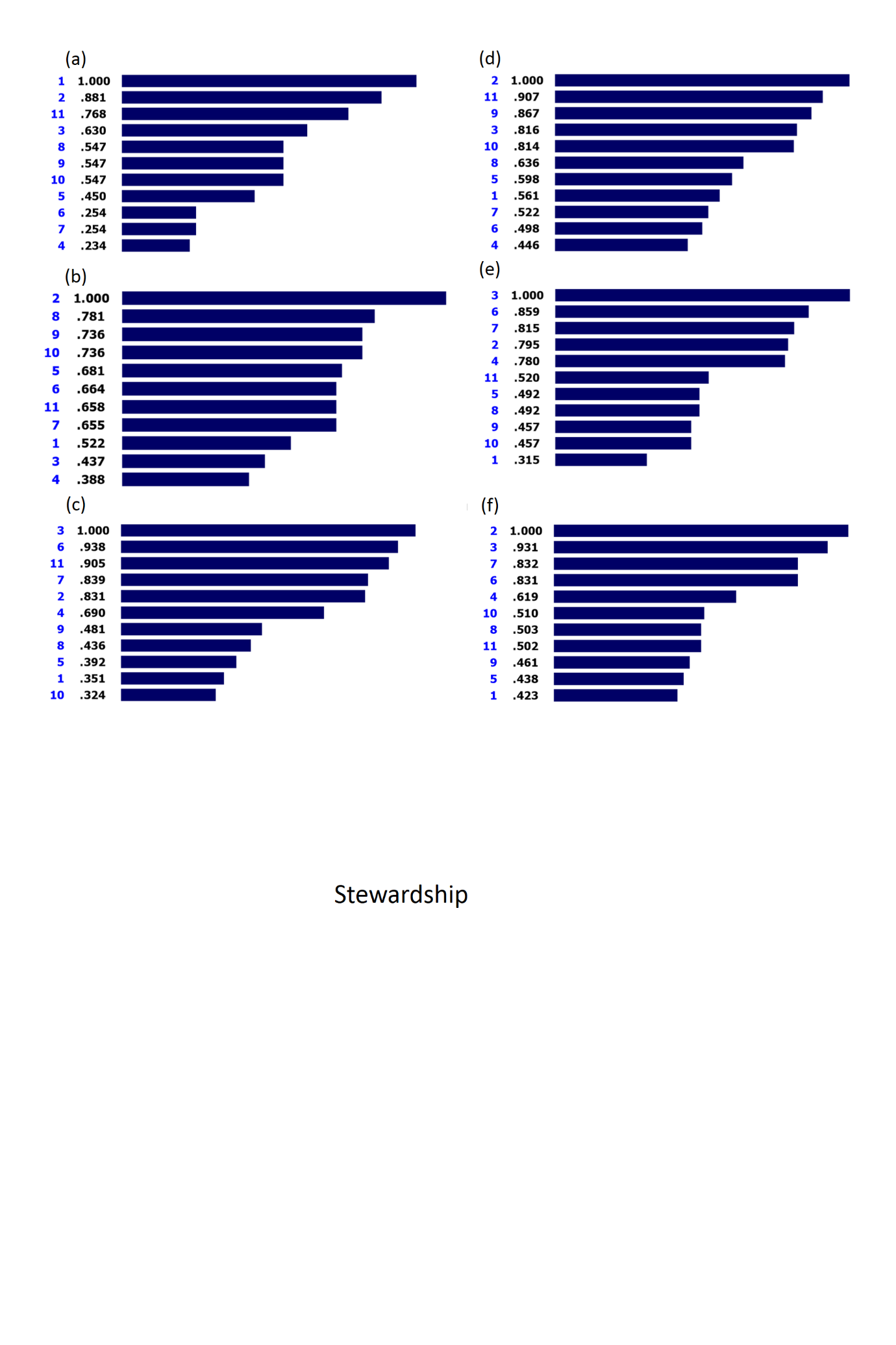


**Supplemental Fig 3** Prioritization of recommendations for collection of funds based on six criteria: (a) effectiveness; (b) acceptability; (c) cost; (d) fairness; (e) feasibility; and (f) time.


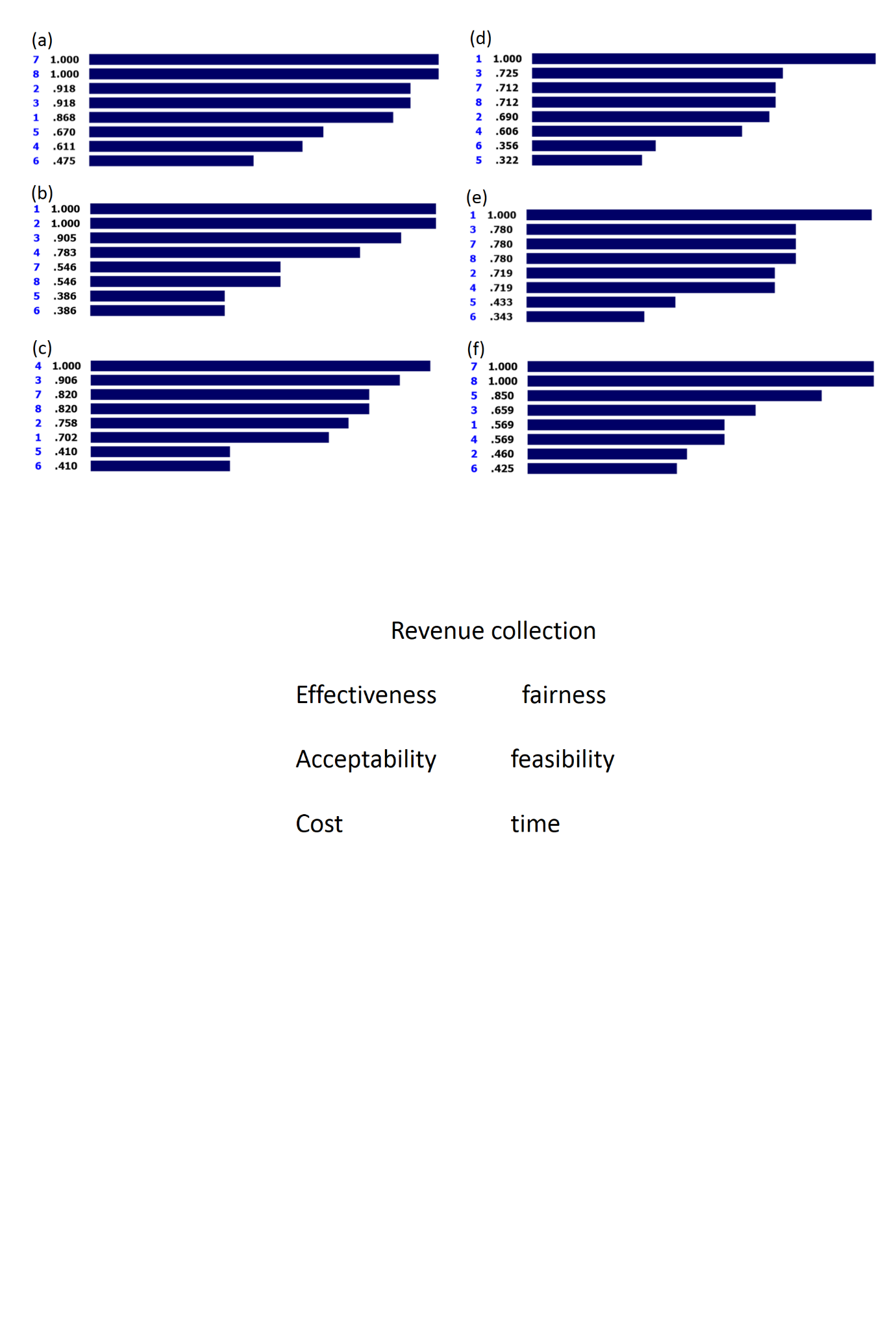


**Supplemental Fig 4** Prioritization of recommendations for pooling of funds based on six criteria: (a) effectiveness; (b) acceptability; (c) cost; (d) fairness; (e) feasibility; and (f) time.


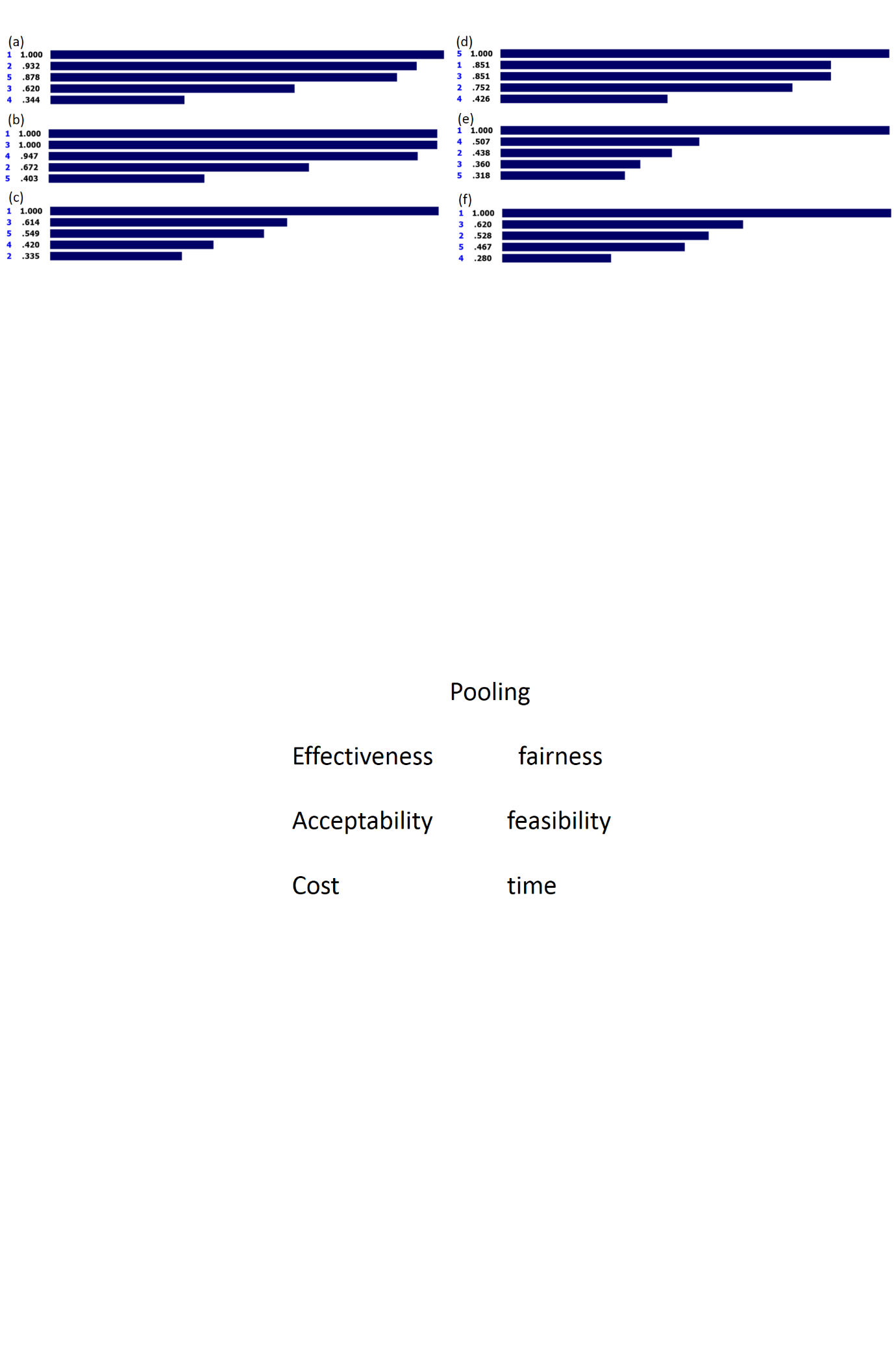


**Supplemental Fig 5** Prioritization of recommendations for purchasing based on six criteria: (a) effectiveness; (b) acceptability; (c) cost; (d) fairness; (e) feasibility; and (f) time.


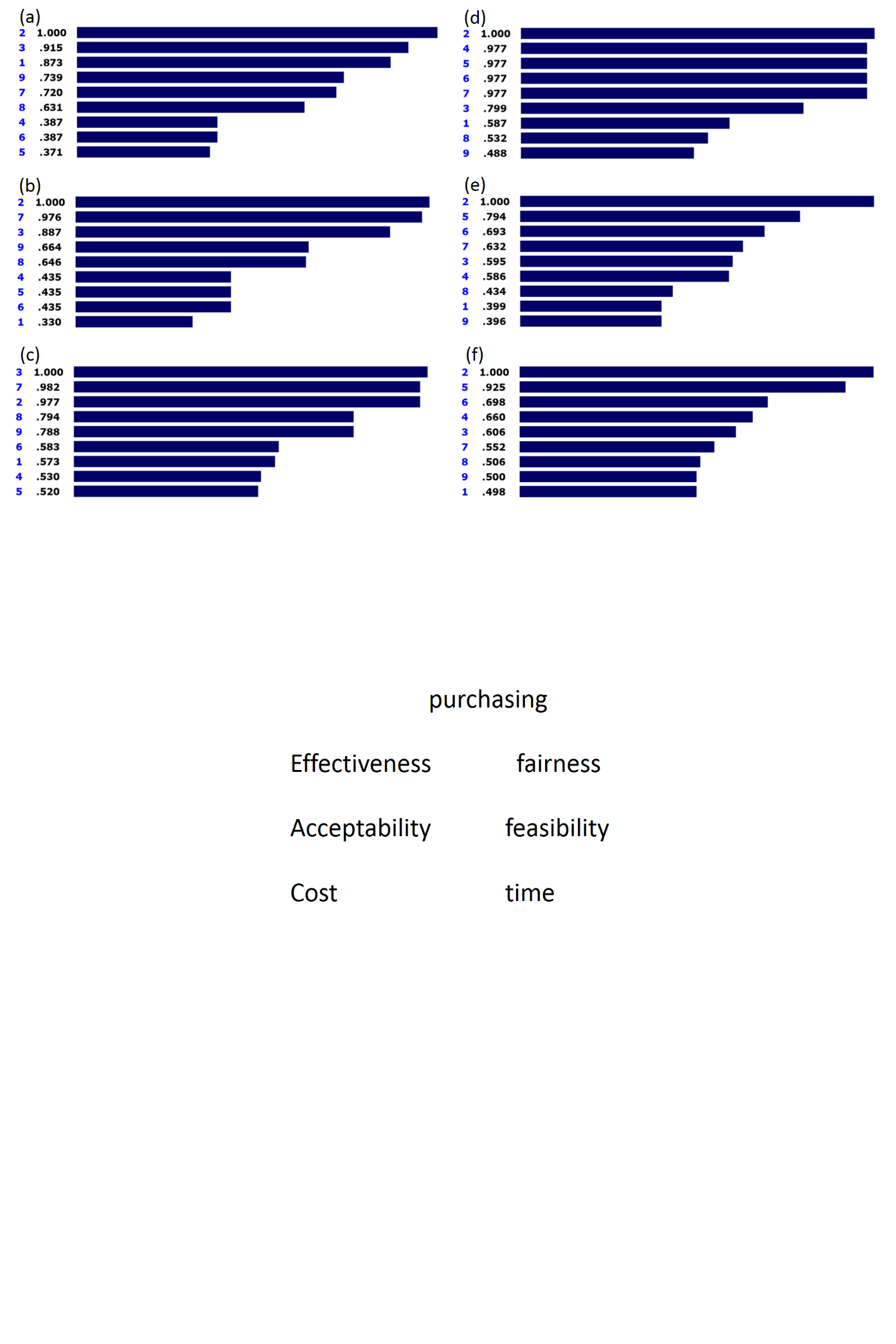


**Supplemental Fig 6** Prioritization of recommendations for benefit package on six criteria: (a) effectiveness; (b) acceptability; (c) cost; (d) fairness; (e) feasibility; and (f) time.


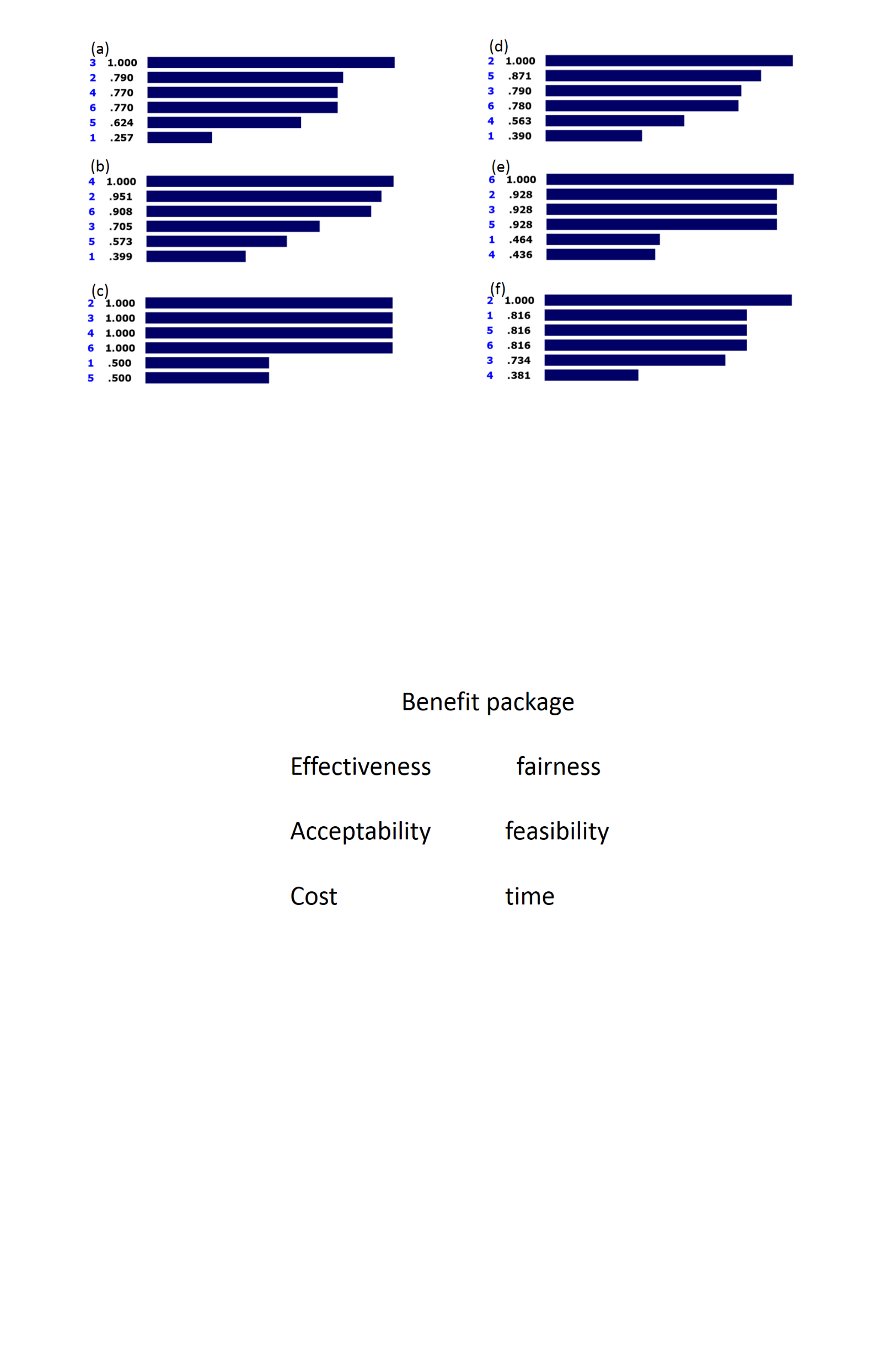

Supplement: Supplementary file 1 — Additional file 1: Figure S1. Conceptual framework. Figure S2. Prioritization of recommendations for stewardship based on six criteria. Figure S3. Prioritization of recommendations for collection of funds based on six criteria. Figure S4. Prioritization of recommendations for pooling of funds based on six criteria. Figure S5. Prioritization of recommendations for purchasing based on six criteria. Figure S6. Prioritization of recommendations for benefit package on six criteria. [file 12962_2021_333_MOESM1_ESM.docx]
